# Acculturation is associated with 12-month adherence to combined MVPA and sedentary behavior guidelines in a sample of midlife and older Latino/a adults: findings from the COMPASS physical activity trial

**DOI:** 10.1186/s12889-025-21885-3

**Published:** 2025-02-22

**Authors:** Astrid N. Zamora, Maria I. Campero, Dulce M. Garcia, Diana M. Chavez, Abby C. King

**Affiliations:** 1 Department of Epidemiology & Population Health, Stanford University School of Medicine, Stanford University, 1701 Page Mill Road, Palo Alto, California 94304 USA; 2 Stanford Prevention Research Center, Department of Medicine, Stanford University School of Medicine, Stanford, CA USA

**Keywords:** Acculturation, Behavioral trial, Latino/a, MVPA, Midlife adults, Older adults, Physical activity, Sedentary behavior

## Abstract

**Background:**

Although some studies have shown that greater acculturation is associated with an increased risk of cardiovascular disease (CVD) among Latino/a adults, the relationship between acculturation and modifiable health behaviors in older Latino/a adults living in the United States (US) remains underexplored. This secondary analysis of the COMPASS physical activity trial explored the relationship between acculturation and adherence to combined movement behavior guidelines for moderate to vigorous physical activity (MVPA) and sedentary behavior guidelines at 12 months among 245 midlife and older Latino/a adults from the San Francisco Bay Area.

**Methods:**

Baseline acculturation was measured using the Short Acculturation Scale for Hispanics, yielding a total score and three subscales on a 5-point Likert scale, higher scores represented higher acculturation. MVPA was evaluated via the Community Healthy Activities Model Program for Seniors (CHAMPS) questionnaire, while sedentary behavior was measured using a validated 1-week recall survey. We employed logistic regression to analyze linear and non-linear associations between acculturation and adherence to the combined MVPA (≥ 150 min/wk) and sedentary behavior (< 8 h/day) movement behavior guidelines, adjusting for baseline MVPA and sedentary behavior, intervention arm, gender, income, body mass index, education, and years in the US.

**Results:**

At baseline, 6.4% adhered to combined MVPA and sedentary behavior guidelines, with the prevalence of adherence rising to 30.2% at 12 months. Each one-point increase in language use acculturation subscale score was linked to 1.7 times greater adherence (*p* = 0.01) at 12 months. A similar pattern was observed when acculturation was operationalized as a binary variable (*p* = 0.03). For the summary acculturation score, participants with higher acculturation had 2.6 times higher odds of adhering to guidelines at 12 months compared to those with lower acculturation (*p* = 0.04).

**Conclusions:**

Higher acculturation was associated with a higher odds of 12 month adherence to MVPA and sedentary behavior guidelines among a sample of older Latino/a adults. Results underscore the importance of incorporating acculturation into behavioral trialsand the need to tailor physical activity interventions for Latino/a older adults with lower acculturation.

**Trial registration:**

Clinicaltrials.gov NCT02111213 Registered April 2, 2014 https//clinicaltrials.gov/study/NCT02111213.

## Background

Cardiovascular disease (CVD) is the leading cause of death in the United States (US) [1]. A 2021 report revealed that the prevalence of CVD among US-born and foreign-born Latino/a adults, was 43% for women and 52% for men [2]. However, given the evolving nature of health trends and the impact of factors such as rising obesity rates and the COVID-19 pandemic [3], these prevalence rates may be much higher today. Common behavioral risk factors for CVD include but are not limited to lifestyle behaviors, for example poor diet quality), physical inactivity, long-term or chronic tobacco use, and alcohol consumption [4]. Specifically, amonga adults of Latin/Hispanic descent, results from the Hispanic Community Health Study/Study of Latinos (HCHS/SOL) revealed that 71% of women and 80% of men had at least one risk factor for CVD [5]. While modifiable risk factors of CVD have been widely explored [6–8], most existing studies have lacked a focus on contextual factors, such as acculturation, that could be contributing to CVD..

Acculturation– the process by which individuals or groups adopt or adapt to different cultural practices, values, or norms– often involves significant shifts in health behavior, language preferences, and social customs [9]. These shifts typically align with the behaviors and lifestyle patterns of the population they have joined, which can influence various aspects of health [9]. For example, as individuals acculturate to US society, they may adopt dietary patterns and behaviors that are more common in the general population, often leading to adverse health outcomes [10]. These shifts have been linked to changes in diet, anxiety, depression, chronic disease, and substance use among both US-born or foreign-born minority populations [11]. For instance, a study among Latino/a women living in the US found that as women became more acculturated, their diet quality worsened, and their sedentary behavior increased, which was associated with a higherrisk of developing diabetes [12]. This association highlights the link between acculturation and modifiable CVD risk factors, which may be addressed through intervention.

While existing studies have explored the links between acculturation and a variety of health behaviors in the general Latino/a adult population, the relationship between physical activity and sedentary behavior among midlife (ages 41–64 years) and older (ages 65 +) Latino/a adults remains less understood. Regular physical activity (PA) and reducing sedentary behavior are critical for safeguarding the health of aging adults, serving as key components of health promotion efforts [13]. Furthermore, it is imperative to recognize that these behaviors are interconnected, with changes in one behavior likely influencing the other (e.g., as physical activity increases, sedentary behavior decreases) [14]. Therefore, a comprehensive examination of both PA and sedentary behavior in relation to acculturation is critical within this demographic of aging Latino/a adults in the US. To address the gap in the current literature, we conducted aw secondary analysis of the Computerized Physical Activity Support for Seniors (COMPASS) trial. This longitudinal analysis examined whether baseline acculturation levels were associated with adherence to combined moderate-to-vigorous physical activity (MVPA) and sedentary behavior guidelines over 12 months among a sample of US-born and foreign-born midlife and older Latino/a adults residing in the San Francisco Bay Area.

## Methods

### Study design and participants

This secondary analysis involved participants from the Computerized Physical Activity Support for Seniors (COMPASS) trial– a single-blind, cluster-randomized noninferiority parallel trial conducted by the Stanford University School of Medicine and Northeastern University. The trial received approval from the Institutional Review Board at Stanford University. Participants provided written consent in their chosen language (English or Spanish). The study adhered to the reporting standards outlined in the Consolidated Standards of Reporting Trials (CONSORT) guidelines, and a CONSORT flow diagram detailing participant flow was previously published [15]. Atotal of 94.3% of participants (231 out of 245) completed the 12-month data collection for walking minutes per week. Specifically, 95.1% of participants in the virtual advisor group and 93.4% in the human peer advisor group completed the data collection. For participants with missing data, imputation methods were used to account for the missing values, ensuring the robustness of the analyses. Complete methods for imputing missing values have been previously described elsewhere [15].

Participants were enrolled in the trial between July 2014 and July 2016 using geographically defined targeted mailings, cultural media-based promotion, and community outreach. The trial’s final 12-month visit occurred in September 2017. Participants were recruited from local community centers in Santa Clara and San Mateo counties (California, US). Each community center was randomized in pairs (1:1 allocation) based on locale to either virtual or human PA advisors based on a computerized randomization sequence. Both arms received a similar 12-month behavioral PA instruction/support program at their designated center based on Active Choices—an individually tailored health behavior program with demonstrated effectiveness and translatability [15]. The interventions focused on walking and similar moderate-intensity PA, which has been shown to have health benefits, especially in older populations [15], with the main goal being to increase total walking and MVPA minutes perweek. The virtual advisor, described in detail elsewhere [16, 17], was an interactive, embodied conversational agent simulating face-to-face counseling using simple speech (synthetic English or Spanish) and non-verbal behaviors (e.g., facial cues and hand gestures), which had been shown to be effective in increasing walking behavior in an older Latino/a sample [17, 18]. Criteria for the trained human peer advisors and complete study methods have been previously described [15, 16].

### Acculturation assessment

Acculturation was assessed via the 12-item Short Acculturation Scale for Hispanics (SASH) [19], with subscales assessing participants’ language use (5 items related to the language spoken and thought), media preference (3 items regarding the language of television programs watched), and social/ethnic relations (4 items assessing the ethnicity of close friends and visitors). The language use subscale evaluates participants’ preference and frequency of using Spanish versus English across various contexts, including speaking, thinking, and reading. It captures the extent to which individuals have adopted the dominant language of the host culture..This subscale uses a 5-point Likert scale to assess language use, the scale ranges from 1 to 5, where: 1 indicates "only Spanish", while 5 indicates preference for "only English." A higher score on this subscale indicates a greater preference for and use of English in these contexts.The media preference subscale assesses the language of television and radio programs the participants watch or listen to, which reflects their cultural engagement through media. The same 5-point Likert scale used for language prefernece is used for media preference, with a higher score on this subscale indicates a preference for English-language media, suggesting greater integration into mainstream US culture. The social/ethnic relations subscale examines the ethnicity of participants’ close friends and the people they interact with most frequently, providing insight into the social integration and ethnic ties of the individual. This subscale uses a 5-point Likert scale to assess the ethnicity of participants' close friends and the individuals they interact with most frequently. The scale ranges from 1 to 5, where: 1 indicates that all of the participant's close friends and frequent interactiosn are with Latino/Hispanics, while 5 indicates that all of the participant's close friends and frequent interactions are with Americans (non-Latino/Hispanic). Ahigherscorereflects more interaction with American (non-Latino) individuals and captures the degree of social integration with the mainstream US culture [5, 19].. [, We created a mean acculturation summary score for all 12 items, with scores ranging from 1 to 5 and higher scores indicating a higher degree of acculturation. We also created mean scores for each of the three subscales, with scores ranging from 1 to 5. For each subscale, a higher score indicated a higher degree of English language use (language use subscale), social/ethnic relations (social/ethnic relations subscale), and media preference (media preference subscale). Acculturation was also operationalized as a binary variable [high acculturation level vs. low acculturation level (reference group)]. This binary variable was created by using the analytic sample’s mean summary score of 3.0 as the cut-point. Participants with a score greater than or equal to the sample mean were dichotomized into the high acculturation level group, while those below the sample mean were dichotomized into the low acculturation group. Cut-points (i.e., sample means) for language use, social/ethnic relations, and media preference subscales were 2.9, 2.7, and 3.4, respectively. Participants with scores at or above these cut-points were categorized into the high acculturation group for each respective subscale, while those with scores below these cut-points were placed in the low acculturation group for each subscale.

###  MVPA and sedentary behavior assessment

For this secondary analysis, the primary outcome of interest was 12-month adherence to two of the three 24-hour movement behavior guidelines - specifically moderate-to-vigorous physical activity (MVPA) and sedentary behavior. For MVPA, we used guidelines from the US Department of Health and Human Services, specifically adherence to ≥ 150 minutes per week of MVPA [20]. Although the major health US and global health organizations, such as the Centers for Disease Control, the American Heart Association, and the World Health Organization, do not specify a limit for sedentary behavior, existing research has shown that  ≥ 8 hours perday of sedentary time is associated with increased risk of premature mortality [21]. Therefore, we used this threshold for sedentary behavior adherence, with less than 8 hours per day indicating adherence to this behavior. An overall adherence value of 1 was assigned for meeting both criteria, and 0 for failing to meet either of them. MVPA levels were assessed using theCommunity Healthy Activities Model Program for Seniors (CHAMPS) PA questionnaire [22], a validated tool known for accurately capturing physical activity levels among older adults. We opted against using device-based tools like accelerometers due to the lack of normative data specifically for older adults, which limits the ability to accurately compare accelerometer-derived physical activity data with established guidelines for this demographic [15]. The absence of appropriate reference values for older adults made it challenging to interpret the results in a meaningful way within the context of physical activity recommendations, leading us to select alternative methods for assessing physical activity. Sedentary behavior was evaluated usinga validated 1-week recall survey [23], with the results converted to the equivalent of 56 hours per week to align with the sedentary behavior guideline of < 8 hours per day.

### Statistical analysis

First, we examined differences in baseline sociodemographic factorsacculturation scores and intervention arm assignment based on whether participants were adherentor non-adherent to MVPA and sedentary behavior guidelines at 12 months. The chi-square or Fisher's chi-square test were employed to analyze differences in all binary and categorical variables, while t-tests were utilized for continuous variables.

Next, we employedlogistic regression models to examine associations between acculturation and odds of adhering to the combined MVPA and sedentary behavior guidelines at 12 months. In the first model, we included acculturation scores (summary and subscales) as continuous variables,adjusting for baseline adherence to guidelines and intervention arm assignment. This model aimed to quantify the crude association between continuous acculturation and adherence to both guidelines. In the second model, we included baseline adherence to MVPA and sedentary behavior, intervention arm assignment, and additionalcovariates that have been shown to be confounders in studies examining acculturation and other health behaviors, including gender [24], income [25], body mass index (BMI) [25], education [25] and years in the US [26, 27]. To measure years in the US, participants were asked how many years they had lived in the country. For individuals born in the US, the number of years in the US was treated as their age at the time of study. For participants born outside of the US, the number of years they had lived in the US was recorded as a continuous variable, rounded to the nearest year. We treated this variable as a continuous predictor in the regression model.

Next, we examined potential non-linear associations between acculturation and odds of adherence at 12 months. We ran separate logistic regression models whereby acculturation (summary score and subscale scores) was modeled as a binary variable, with the low acculturationgroup serving as the reference for all models. For these analysis, the first model adjusted for baseline adherence to MVPA and sedentary behavior, while the second model adjusted for the same covariates included in the adjusted model where acculturation was treated as a continous measure.

For all models, the odds ratios (OR) and corresponding 95% confidence intervals (CIs) are reported. Statistical tests were two-sided, with significance set at *P* < 0.05. All statistical analyses were conducted using SAS 9.4 (SAS Institute Inc., Cary, NC).

## Results

The study included 245 participants with a mean (SD) age of 62.3 (8.4) years across the overall sample, with 78.8% identifying as women. At baseline, 6.4% adhered to the guidelines, while 30.2% adheredr at 12 months. A detailed description of baseline participant characteristics is provided in Table 1. We observed marginal differences in participants’ age according to adherenceo to guidelines at 12 months, with those who did not adherebeing slightly older than those who adhered [62.9 (8.7) years vs. 60.8 (7.3) years, *p* = 0.06]. Additionally, we observed differences in years spent in the US based on 12 month adherence status. Specifically,those who adhered to guidelines at 12 months had a mean (SD) time in the US of 41.3 (16.2) years compared to 50.1 (16.7) years for those who did not adhere (*p* = 0.0002). Participants were born in over 13 different countries across Latin America and the US. A statistically significant difference(*p *= 0.03) in the distribution of country of birth between those who adhered to the guidelines and those who did not adhere. A larger proportion of participants born in Mexico adhered to the guidelines (12.7%), while a significantly higher proportion of participants born in the US did not adhere (32.7%) compared to US born participants who adhered (8.9%). No significant differences wereobserved for the acculturation summary score or subscale scores according to adherence at 12 months.The mean (SD) acculturation summary score for the entire sample was 3.0 (1.1), while mean (SD) scores for subscales ranged from 2.7 (0.7) to 3.4 (1.4). No other statistical differences were observed.

**Table 1:** Baseline characteristics of COMPASS trial participants stratified by adherence to MVPA and sedentary behavior guidelines at 12 months (*N* = 245)

	Overall sample Mean (SD) or % (*N*)	Not adhering to guidelines at 12 months (*n* = 171) Mean (SD) or %	Adhering to guidelines at 12 months (*n* = 74) Mean (SD) or %	*P*
Age, years, mean (SD)	62.3 (8.4)	62.9 (8.7)	60.8 (7.3)	0.06
Gender, N (%)				
*Women*	78.8 (193)	56.3	22.5	0.26
*Men*	21.2 (52)	13.5	7.8	
Study arm, N (%)				
*Virtual advisor*	50.2 (123)	35.9	14.3	0.54
*Human peer advisor*	49.8 (122)	33.9	15.9	
Education, years, mean (SD)	12.8 (4.1)	12.8 (3.8)	12.7 (4.9)	0.76
US or Foreign-born				
*US-born*	41.6 (102)	32.5	21.6	**0.01**
*Foreign-born*	58.4 (143)	37.1	21.2	
Country of birth, N (%)				
*Bolivia*	2 (0.8)	0.0	0.8	**0.03**
*Chile*	1 (0.4)	0.0	0.4	
*Colombia*	2 (0.8)	0.4	0.4	
*Ecuador*	1 (0.4)	0.0	0.4	
*El Salvador*	25 (10.2)	6.9	3.3	
*Guatemala*	2 (0.8)	0.4	0.4	
*Honduras*	2 (0.8)	0.4	0.4	
*Mexico*	87 (35.5)	22.9	12.7	
*Nicaragua*	14 (5.7)	0.0	0.4	
*Panama*	1 (0.4)	0.0	0.4	
*Peru*	2 (0.8)	0.4	0.4	
*Puerto Rico*	1 (0.4)	0.4	0.0	
*US*	102 (41.6)	32.7	8.9	
Unknown country of birth, not US-born*	3 (1.2)	1.2	0.0	
Time in the US, years, mean (SD)	47.4 (17.0)	50.1 (16.7)	41.3 (16.2)	**0.0002**
Marital status, N (%)				
*Married or partnered*	51.0 (125)	37.9	13.1	0.46
*Not married or partnered*	10.2 (25)	6.5	3.7	
*Divorced*,* separated*,* or widowed*	31.8 (78)	20.8	11.0	
*Refused*,* other*,* missing*	6.9 (17)	4.5	2.5	
Household size, mean (SD)	3.5 (2.2)	3.5 (2.3)	3.4 (2.1)	0.60
Income, N (%)				
*<$ 5*,*000 - $34*,*999*	13.1 (32)	7.4	5.7	0.23
*$35*,*000 - $49*,*999*	9.4 (23)	6.1	3.3	
*$50*,*000 - $74*,*999*	12.2 (30)	7.8	4.5	
*> $75*,*000*	45 (18.4)	14.3	4.1	
*Refused*,* does not know*,* or missing*	46.9 (115)	34.3	12.7	
BMI, mean (SD)	33.2 (8.3)	33.6 (8.6)	32.0 (7.6)	0.17
Vitality plus score, mean (SD)	34.6 (7.9)	34.4 (8.1)	35.1 (7.7)	0.50
SASH summary score, mean (SD)	3.0 (1.1)	3.0 (1.1)	2.8 (1.1)	0.12
SASH language use subscale, mean (SD)	2.9 (1.4)	3.0 (1.4)	2.7 (1.3)	0.10
SASH media preference subscale, mean (SD)	3.4 (1.4)	3.5 (1.4)	3.3 (1.4)	0.16
SASH social/ethnic subscale, mean (SD)	2.7 (0.7)	2.7 (0.7)	2.6 (0.8)	0.36

*Denotes participants not born in the US that did not provide information on country of birth. Abbreviations: BMI: body mass index; MVPA: moderate-vigorous physical activity; N = sample size; SASH: Short Acculturation for Hispanic Survey; SD: standard deviation; US: United States; All *P*-values from t-tests (continuous variables) or chi-square or Fisher’s exact chi-square test (binary or categorical variables); Note: boldface indicates *p* < 0.05

In Table 2, we present results from models examining the continuous acculturation scores and odds of adhering to MVPAand sedentary behavior guidelines among the sample of midlife and older adults. Results from model 1, which adjusted for adherence to baseline MVPA and sedentary behavior as well as the intervention arm assignment, revealed no significant association between the acculturation summary score or acculturation subscales with adherence to guidelines. However, when we adjusted for potential confounders, we found that every 1-point score increase in the acculturation language use subscale was significantly associated with 1.7 [OR = 1.7 (95% CI: 1.1, 2.6) *p* = 0.01] times higher odds of adhering to the guidelines at 12 months. After full adjustment, no significant associations were observed between the acculturation summary score or the social/ethnic and media preference subscales with odds of adhering to guidelines at 12 months.

**Table 2:** Continuous acculturation and odds of adhering to MVPA and sedentary behavior guidelines among midlife and older adults (*N* = 245)

	Model 1		Model 2	
	OR (95% CI)	*P*	OR (95% CI)	*P*
Acculturation summary score	0.8 (0.6, 1.0)	0.08	1.6 (0.9, 2.6)	0.06
Acculturation language use subscale	0.8 (0.7, 1.0)	0.07	1.7 (1.1, 2.6)	**0.01**
Acculturation social/ethnic subscale	0.8 (0.5, 1.2)	0.22	1.1 (0.7, 1.9)	0.62
Acculturation media preference subscale	0.9 (0.7, 1.1)	0.13	1.2 (0.9, 1.7)	0.19

Model 1: Only adjusted for baseline MVPA and sedentary behavior and intervention arm; Model 2: Adjusted for the same covariates from model 1 + gender, income, BMI, education, and years in the US; ABBR: MVPA: moderate-vigorous physical activity; sedentary behavior: sedentary behavior; OR: Odds ratio; 95%CI: 95% confidence interval; note: boldface indicate *p* < 0.05

**Fig. 1:**
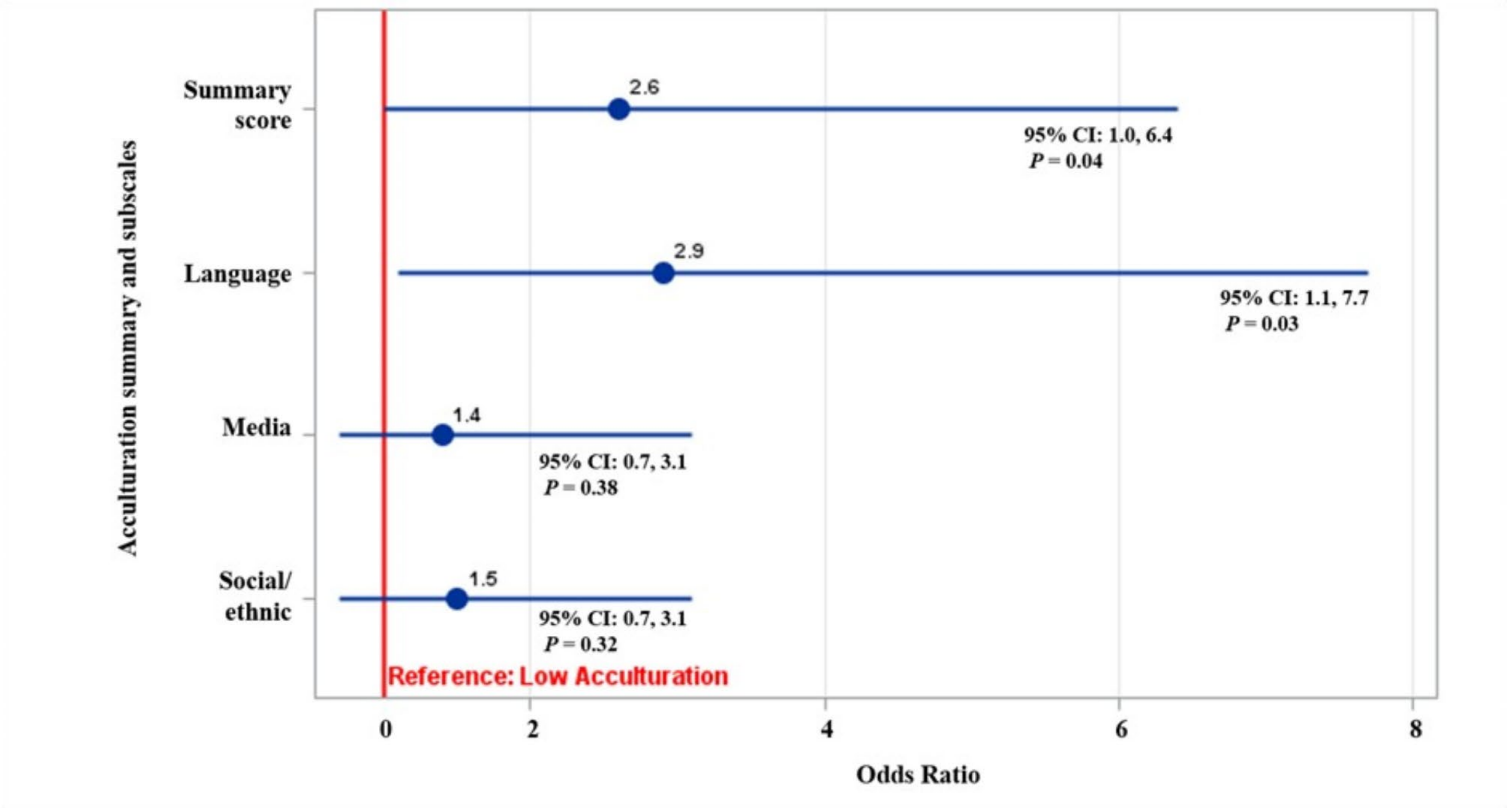
Forest plot of the adjusted association [1] between acculturation groups (low vs. high) and odds of adhering to MVPA + sedentary behavior guidelines at 12 months

Table 3 presents the results from the unadjusted multivariate logistic regression analyses, which focused on examining the association between acculturation groups (low vs. high) and adherence to guidelines at 12 months. Model 1, adjusted for baseline MVPA and sedentary behavior adherence and intervention arm assignment, revealed no significant associations between acculturation groups (low vs. high) for the summary score nor for the acculturation subscales with odds of adhering to guidelines at 12 months. However, after full adjustment for confounders (model 2), results showed that compared to the low acculturation group, those in the high acculturation group had 2.6 [OR (95% CI) = 2.6 (1.0, 6.4); *P* = 0.04] times higher odds of adhering to the MVPA and sedentary behavior guidelines at 12 months (See Fig. 1 for forest plot). Further, whenexamining associations between the low vs. high acculturation groups for the three acculturation subscales and adherence to guidelines, we found that compared to thelow acculturation group for the language use subscale, those in the highacculturation group had 2.9 [OR (95% CI) = 2.97 (1.1, 7.7); *P* = 0.03] times higher odds of adhering to the MVPA and sedentary behavior guidelines at 12 months. No statistically significant associations were observed for the social/ethnic subscale or media preference subscales, either before or after adjusting for potential confounders.

**Table 3:** Unadjusted association between acculturation group (low vs. high) and odds of adhering to MVPA and sedentary behavior guidelines among midlife and older adults (*N* = 245)

	Model 1	
	OR (95% CI)	*P*
Low acculturation summary score (REF)		
High acculturation summary score	0.6 (0.3, 1.1)	0.08
Low acculturation language use subscale (REF)		
High acculturation language use subscale	0.6 (0.3, 1.0)	0.06
Low acculturation social/ethnic subscale (REF)		
High acculturation social/ethnic subscale	0.8 (0.5, 1.4)	0.45
Low acculturation media preference subscale (REF)		
High acculturation media preference subscale	0.6 (0.3, 1.0)	0.06

Model 1: Only adjusted for baseline MVPA and sedentary behavior and intervention arm; Model 2: Adjusted for the same covariates from model 1 + gender, income, BMI, education, and years in the US; ABBR: MVPA: moderate-vigorous physical activity; sedentary behavior: sedentary behavior; OR: Odds ratio; 95%CI: 95% confidence interval; REF = reference group; Acculturation was dichotomized based on the analytic sample’s mean summary score of 3.0, with participants scoring ≥ 3.0 classified as high acculturation and those < 3.0 as low acculturation. For the subscales, cut-points were 2.9 (language use), 2.7 (social/ethnic relations), and 3.4 (media preference). Participants scoring at or above these cut-points were categorized into the higher acculturation group for each subscale, while those below were placed in the lower acculturation group. Note: boldface indicates *p* < 0.05

## Discussion

The present study findings provided evidence of both linear and non-linear associations between acculturation and adherence to combined moderate-to-vigorous physical activity (MVPA) and sedentary behavior guidelines at 12 months among a sample of midlife and older Latino/a adults enrolled in a behavioral physical activity trial. The analysis, which considered acculturation as both a continuous and binary measure, provides valuable insights into the relationship between language use acculturationlevels and adherence to movement behavior guidelines.

Our study found that participants with higher acculturation had nearly three times the odds of adhering to the combined age-specific movement guidelines for MVPA and sedentary behavior at the 12-month study endpoint compared to those with lower acculturation. This finding is generally consistent with previous research linking acculturation levels to physical activity (PA) and sedentary behavior [28]. For instance, lower acculturation, often reflecting limited cultural adaptation to the dominant culture, have often been associated with lower levels of PA [28] as well as reduced sedentary time among Latinas [29]. On the other hand, greater acculturation has been linked to higher levels of MVPA [30] and a greater likelihood of meeting PA guidelines [31], with similar patterns observed primarily in women. Our findings align with these trends, suggesting that acculturation influences engagement in physical activity, though the direction and strength of this relationship can vary across studies. However, we caution that comparisons between studies should consider differences in populations, measurement tools, and methodologies. While our study relied solely on self-reported PA and sedentary behavior, previous research has reported mixed results when comparing self-reported versus device-assessed PA. For instance, a nationally representative sample of US men found that although greater acculturation was linked to higher self-reported PA, device-assessed PA actually decreased with acculturation [32]. Similarly, a study of Mexican adults found that acculturation measures were associated with self-reported sitting but not with accelerometer-assessed sedentary behavior [33]. These discrepancies highlight the importance of considering measurement techniques in future research. Importantly, to our knowledge, no studies have yet explored the relationship between acculturation and adherence to movement behavior guidelines among Latino/a midlife and older adults residing in the US. Therefore, our work helps fill a gap in the the current literature by examining how differences in acculturation levels relate to adherence to these guidelines, offering valuable insights into this underexplored area.

Various aspects of acculturation have been examined in relation to movement behaviors [33–35], highlighting the nuanced and multifaceted nature of this process. While individuals may demonstrate higher acculturation in language proficiency or preference, their acculturation levelin other domains, suchassocial/ethnicrelations[33], may avary and could be differentially related to movement behavior outcomes. a For example, in a study of Mexican adults from the HCHS/SOL study, English-language preference was associated with leisure time and accelerometer-based MVPA [33]. In contrast, other studies have indicated that nsocial/ethnic relations measures of acculturation are less predictive of movement behaviors [36]. Compared to existing research, our study revealed that every one-point increase in the language use acculturation subscale score was associated with nearly two times higher odds of adhering to the combined MVPA and sedentary behavior guidelines at 12 months. Moreover, when acculturation was modeled as a binary measure (low vs. high based on scores), the model revealed a stronger association between acculturation and adherence to movement behaviorguidelines compared to the model with acculturation operationalized as a continous measure. This intriguing finding may be attributed to several factors that merit consideration. One possible explanation for the stronger binary association could be related to the categorical nature of the acculturation measure. By categorizing acculturation scores into high vs. low groups based on language use, we potentially captured distinct cultural identities and behavioral patterns that align more directly with adherence to movement behaviorguidelines. Furthermore, language use may serve as a more immediate and visible marker of acculturation, influencing behaviors such as physical activity by providing greater access to resources, social networks, and cultural norms that promote physical activity. In contrast, the social/ethnic and media preference subscales may reflect more complex cultural dynamics that are less directly tied to movement behaviors. These results highlight the importance of considering different facets of acculturation when examining health behaviors in diverse populations.

The findings from our study contribute to the ongoing discussion in acculturation research, which often associates greater acculturation with poorer lifestyle behaviors, such as diet [31] and substance use [37]. In contrast, our results revealed that higher acculturation scores wereassociated with greater adherence to self-reported physical activity and sedentary behavior guidelines, a finding that is consistent with previous research showing that acculturation is positively associated with physical activity levels among Hispanic and Latinoadults [33]. While these findings align with existing literature on self-reported physical activity, they differ from the observed patterns in diet-related behaviors, where greater acculturation is often linked to poorer dietary habits. This distinction underscores the complex and multifaceted nature of acculturation and its differential effects on health behaviors. These results emphasize the need forfurther exploration of how physical activity or lower sedentary behavior may serve as protective factors for individuals with higher levels of acculturation, particularly in Latino/a midlife and older adult populations. Understanding the interplay between acculturation, physical activity, and sedentary behavior can inform the development of targeted interventions to promote active lifestyles and reduce health risks in this underserved demographic.

Moving forward, it is crucial to consider the nuances of acculturation when designing interventions for Latino/a populations. Given the varying levels of acculturation within this group, interventions should be tailored to address specific cultural and behavioral patterns. For example, interventions for individuals with higher levels of acculturation might focus on reinforcing existing engagement with physical activity, while those with lower levels of acculturation may benefit from bridging cultural gaps, such as incorporating culturally relevant physical activity norms or improving access to resources that promote active lifestyles. Furthermore, it will be critical to understand how acculturation-related factors, such as language preference or social ties to cultural communities, influence participants’ motivation and adherence to movement behavior guidelines. By considering these cultural factors, future interventions can be more effective in fostering sustainable behavior change and improving health outcomes in Latino/a midlife and older adults.

### Strengths and limitations

This secondary study demonstrates several strengths. First, it fills a notable research gap by investigating the relationship between acculturation and adherence to moderate-to-vigorous physical activity (MVPA) and sedentary behavior guidelines among midlife and older Latino/a adults in the US, an area of research within a demographic that remains largely understudied. Second, by utilizing longitudinal data from a behavioral trial, we were able to examine changes in participants’ movement behaviors over time, which strengthened our ability to assess the temporal relationship between acculturation and physical activity behaviors. Additionally, the sample inclusively represented Latino/a adults who were born in the US, along with more than half that were born in Mexico, Central and South America, contributing to the heterogeneity within the Latino/a demographic. Nevertheless, our study also faced certain limitations. The study population, although comprising a sample of aging Latino/a adults who are not typically the focus of physical activity trials, tended to be more educated and predominantly female, possibly limiting the generalizability of our findings to the broader demographic and behavioral profiles of Latino/a midlife and older adults residing in the US. Furthermore, the assessment of MVPA and sedentary behavior relied on self-reported measures, despite the use of validated instruments such as a 1-week recall and the CHAMPS questionnaire. Moreover, the direction of the association between acculturation level and self-reported MVPA may differ from the direction of the association between acculturation and objectively assessed MVPA, thus shedding light on the need for including objective measures to provide more comprehensive estimates and validity to the directionality of the observed associations. While using self-reported instruments offers advantages in terms of accessibility and participant reach, the inherent risk of recall bias in self-reporting underscores the need for incorporating objective measures in future research to provide a more robust evaluation of movement behaviors. Finally, our findings focused only on adherence or non-adherence to moderate-intensity aerobic activity and did not include adherence to balance or muscle-strengthening activities, which are also included in US physical activity guidelines. This represents a limitation of the current study, as it does not fully align with the broader 24-hour movement behavior guidelines, which emphasize both aerobic and muscle-strengthening activities. Future research should explore the role of balance and muscle-strengthening exercises in older adults, given their importance in maintaining overall health and functionality and their inclusion in physical activity guidelines.

## Conclusion

In this physical activity trial among midlife and older Latino/a adults from the San Francisco Bay Area, we found that higher baseline acculturation levels were associated with greater odds of adhering to MVPA and sedentary behavior guidelines at the 12-month study endpoint, regardless of intervention armassignment. These results underscore the importance of integrating acculturation considerations into the design of behavioral trials and highlight the need to of tailor physical activity interventions specifically for aging Latino/a adults with lower acculturation.

## Data Availability

The dataset analyzed during the current study is not publicly available. However, upon reasonable request, the corresponding author (astridz@stanford.edu) can provide data access.

## References

[1] Kochanek KD. Mortality in the United States, 2022. 2024;(492).

[2] Virani SS, Alonso A, Aparicio HJ, Benjamin EJ, Bittencourt MS, Callaway CW, et al. Heart Disease Stroke Statistics—2021 Update Circulation. 2021;143(8):e254–743. 33501848 10.1161/CIR.0000000000000950 PMC13036842

[3] Sun M, Yuan M, Lai H, Wang Q, Wang H, Xing L, et al. Increased risk of new-onset cardiovascular disease after COVID-19: a systematic review and meta-analysis of 14 cohorts. Rev Med Virol. 2024;34(2):e2518.

[4] Cardiovascular diseases (CVDs). [cited 2024 Apr 22]. Available from: https://www.who.int/news-room/fact-sheets/detail/cardiovascular-diseases-(cvds).

[5] Hispanic Community Health Study/Study of Latinos (HCHS/SOL).| NHLBI, NIH. [cited 2024 May 23]. Available from: https://www.nhlbi.nih.gov/science/hispanic-community-health-studystudy-latinos-hchssol

[6] Yusuf S, Joseph P, Rangarajan S, Islam S, Mente A, Hystad P, et al. Modifiable risk factors, cardiovascular disease and mortality in 155,722 individuals from 21 high-, middle-, and low-income countries. Lancet Lond Engl. 2020;395(10226):795–808. 10.1016/S0140-6736(19)32008-2 PMC8006904 31492503

[7] Adhikary D, Barman S, Ranjan R, Stone H. A systematic review of Major Cardiovascular Risk factors: a growing Global Health concern. Cureus 14(10):e30119. 10.7759/cureus.30119 PMC9644238 36381818

[8] Global Cardiovascular Risk Consortium, Magnussen C, Ojeda FM, Leong DP, Alegre-Diaz J, Amouyel P, et al. Global effect of modifiable risk factors on Cardiovascular Disease and Mortality. N Engl J Med. 2023;389(14):1273–85. 37632466 10.1056/NEJMoa2206916 PMC10589462

[9] Fox M, Thayer Z, Wadhwa PD. Acculturation and health: the moderating role of socio-cultural context. Am Anthropol. 2017;119(3):405–21. 28966344 10.1111/aman.12867 PMC5617140

[10] Arandia G, Nalty C, Sharkey JR, Dean WR. Diet and Acculturation among Hispanic/Latino older adults in the United States: a review of literature and recommendations. J Nutr Gerontol Geriatr. 2012;31(1):16–37. 22335438 10.1080/21551197.2012.647553

[11] Deslandes C, Kaufmann LM, Anderson JR. The relationship between acculturation and relevant correlates for sub-saharan and north African-born migrants: a meta-analytic review. Int J Intercult Relat. 2024;98:101928.

[12] O’Brien MJ, Shuman SJ, Barrios DM, Alos VA, Whitaker RC. A qualitative study of acculturation and diabetes risk among urban immigrant latinas: implications for diabetes Prevention efforts. Diabetes Educ. 2014;40(5):616–25. 24872386 10.1177/0145721714535992 PMC4169339

[13] Gao Z, Lee JE. Promoting physical activity and reducing sedentary behavior to Prevent Chronic diseases during the COVID Pandemic and Beyond. J Clin Med. 2022;11(16):4666. 36012905 10.3390/jcm11164666 PMC9410464

[14] Higgins S, Pomeroy A, Bates LC, Paterson C, Barone Gibbs B, Pontzer H, et al. Sedentary behavior and cardiovascular disease risk: an evolutionary perspective. Front Physiol. 2022;13:962791. 35965885 10.3389/fphys.2022.962791 PMC9363656

[15] King AC, Campero MI, Sheats JL, Castro Sweet CM, Hauser ME, Garcia D, et al. Effects of counseling by peer human advisors vs computers to increase walking in Underserved populations: the COMPASS randomized clinical trial. JAMA Intern Med. 2020;180(11):1481. 32986075 10.1001/jamainternmed.2020.4143 PMC7522781

[16] King AC, Campero I, Sheats JL, Castro Sweet CM, Garcia D, Chazaro A, et al. Testing the comparative effects of physical activity advice by humans vs. computers in underserved populations: the COMPASS trial design, methods, and baseline characteristics. Contemp Clin Trials. 2017;61:115–25. 28739541 10.1016/j.cct.2017.07.020 PMC5987528

[17] King AC, Bickmore TW, Campero MI, Pruitt LA, Yin JL. Employing virtual advisors in preventive care for underserved communities: results from the COMPASS study. J Health Commun. 2013;18(12):1449–64. 23941610 10.1080/10810730.2013.798374 PMC7187757

[18] Bickmore TW, Pfeifer LM, Byron D, Forsythe S, Henault LE, Jack BW, et al. Usability of Conversational agents by patients with Inadequate Health Literacy: evidence from two clinical trials. J Health Commun. 2010;15(sup2):197–210. 20845204 10.1080/10810730.2010.499991

[19] Marin G, Sabogal F, Marin BV, Otero-Sabogal R, Perez-Stable EJ. Development of a short Acculturation Scale for hispanics. Hisp J Behav Sci. 1987;9(2):183–205.

[20] King AC, Whitt-Glover MC, Marquez DX, Buman MP, Napolitano MA, Jakicic J, et al. Physical Activity Promotion: highlights from the 2018 Physical Activity Guidelines Advisory Committee Systematic Review. Med Sci Sports Exerc. 2019;51(6):1340–53. 31095090 10.1249/MSS.0000000000001945 PMC11002995

[21] Ekelund U, Steene-Johannessen J, Brown WJ, Fagerland MW, Owen N, Powell KE, et al. Does physical activity attenuate, or even eliminate, the detrimental association of sitting time with mortality? A harmonised meta-analysis of data from more than 1 million men and women. Lancet Lond Engl. 2016;388(10051):1302–10. 10.1016/S0140-6736(16)30370-1 27475271

[22] Stewart AL, Mills KM, King AC, Haskell WL, Gillis D, Ritter PL. CHAMPS physical activity questionnaire for older adults: outcomes for interventions. Med Sci Sports Exerc. 2001;33(7):1126–41. 11445760 10.1097/00005768-200107000-00010

[23] Gardiner PA, Clark BK, Healy GN, Eakin EG, Winkler EAH, Owen N. Measuring older adults’ sedentary time: reliability, validity, and responsiveness. Med Sci Sports Exerc. 2011;43(11):2127–33. 21448077 10.1249/MSS.0b013e31821b94f7

[24] Diez Roux AV, Detrano R, Jackson S, Jacobs DR, Schreiner PJ, Shea S, et al. Acculturation and socioeconomic position as predictors of coronary calcification in a multiethnic sample. Circulation. 2005;112(11):1557–65. 16144996 10.1161/CIRCULATIONAHA.104.530147

[25] Divney AA, Echeverria SE, Thorpe LE, Trinh-Shevrin C, Islam NS. Hypertension prevalence jointly influenced by acculturation and gender in US immigrant groups. Am J Hypertens. 2019;32(1):104–11. 30165394 10.1093/ajh/hpy130 PMC6284750

[26] Lee S, Ryu S, Lee GE, Kawachi I, Morey BN, Slopen N. The association of acculturative stress with self-reported sleep disturbance and sleep duration among Asian americans. Sleep. 2022;45(4):zsab298. 34922392 10.1093/sleep/zsab298 PMC8996032

[27] Schwartz SJ, Unger JB, Des Rosiers SE, Lorenzo-Blanco EI, Zamboanga BL, Huang S, et al. Domains of Acculturation and their effects on Substance Use and sexual behavior in recent hispanic immigrant adolescents. Prev Sci. 2014;15(3):385–96. 23828449 10.1007/s11121-013-0419-1 PMC3825845

[28] Delaney CL, Spaccarotella K, Quick V, Byrd-Bredbenner C. A comparison of weight-related behaviors of hispanic mothers and children by Acculturation Level. Int J Environ Res Public Health. 2021;18(2):503. 33435478 10.3390/ijerph18020503 PMC7827543

[29] Perez LG, Chavez A, Marquez DX, Soto SC, Haughton J, Arredondo EM. Associations of Acculturation with Self-Report and Objective Physical Activity and sedentary behaviors among latinas. Health Educ Behav. 2017;44(3):431–8. 27679665 10.1177/1090198116669802 PMC5565158

[30] Santiago-Torres M, Contento I, Koch P, Tsai WY, Gaffney AO, Marín-Chollom AM, et al. Associations between Acculturation and Weight, Diet Quality, and physical activity among latina breast Cancer survivors: the ¡Mi Vida Saludable! Study. J Acad Nutr Diet. 2022;122(9):1703–16. 35398558 10.1016/j.jand.2022.04.002 PMC10030055

[31] Wilkie G, Leung K, Moore Simas TA, Tucker KL, Chasan-Taber L. The Association between Acculturation and Diet and physical activity among pregnant hispanic women with abnormal glucose tolerance. J Womens Health. 2022;31(12):1791–9. 10.1089/jwh.2022.0017 PMC9805839 36040352

[32] Zan H, Fan JX. Reporting more but moving less? The Complex relationship between Acculturation and Physical Activity among US adults. Am J Health Promot. 2018;32(2):446–52. 28660772 10.1177/0890117117716415

[33] Camplain R, Sotres-Alvarez D, Alvarez C, Wilson R, Perreira KM, Castañeda SF, et al. The association of acculturation with accelerometer-assessed and self-reported physical activity and sedentary behavior: the Hispanic Community Health Study/Study of latinos. Prev Med Rep. 2020;17:101050. 32021761 10.1016/j.pmedr.2020.101050 PMC6994298

[34] Evenson KR, Sarmiento OL, Ayala GX. Acculturation and physical activity among North Carolina Latina immigrants. Soc Sci Med. 2004;59(12):2509–22. 15474205 10.1016/j.socscimed.2004.04.011

[35] Inn JTH, Wong BWX, Chan YH, Zhongwei H, Logan SJS, Cauley JA, et al. Associations of reading language preference with muscle strength and physical performance: findings from the Integrated Women’s Health Programme (IWHP). PLoS ONE. 2023;18(4):e0284281. 37036875 10.1371/journal.pone.0284281 PMC10085028

[36] Benitez TJ, Dodgson JE, Coe K, Keller C. Utility of Acculturation in Physical Activity Research in Latina adults: an integrative review of literature. Health Educ Behav. 2016;43(3):256–70. 27178493 10.1177/1090198115601042 PMC12964549

[37] Caetano R, Clark CL. Acculturation, alcohol consumption, smoking, and drug use among hispanics. Acculturation: advances in theory, measurement, and applied research. Washington, DC, US: American Psychological Association; 2003;223–39.

